# Aerosol emission and exposure in non-invasive ventilation

**DOI:** 10.1038/s41598-025-98751-0

**Published:** 2025-04-23

**Authors:** Petra Nikuri, Anthony Maalouf, Ahmed Geneid, Eero Pesonen, Enni Sanmark, Ville A. Vartiainen

**Affiliations:** 1 Heart and Lung Center, Faculty of Medicine, Helsinki University Hospital, University of Helsinki, Helsinki, Finland; 2 Department of Otorhinolaryngology ja Phoniatrics - Head and Neck Surgery, University of Helsinki and Helsinki University Hospital, Helsinki, Finland; 3 Department of Anesthesiology and Intensive Care Medicine, University of Helsinki and Helsinki University Hospital, Helsinki, Finland

**Keywords:** Aerosol emission, High-Flow nasal cannula (HFNC), Continuous positive airway pressure (CPAP), Respiratory aerosols, Health care, Medical research

## Abstract

From the beginning of the COVID-19 pandemic, there has been concern among clinicians whether the use of high-flow nasal cannula (HFNC) and continuous positive airway pressure (CPAP) contributes to aerosol generation and consequently spreading of pathogens. Most guidelines still classify these treatments as high-risk aerosol-generating procedures. The aim of this study was to evaluate differences in aerosol emissions and exposure with CPAP and HFNC compared to no breathing aid (NBA). Aerosol emissions of 16 healthy volunteers using CPAP, HFNC and NBA were measured with a portable aerosol spectrometer. During each measurement, the volunteers were instructed consecutively to breathe normally, breathe deeply, cough and read aloud a predefined text. The Wilcoxon signed-rank test was used in statistical analysis. Non-invasive ventilation (CPAP, HFNC) does not produce significantly more aerosol than the same respiratory activities without a breathing aid (median CPAP-NBA − 4.54 1/L, *p* = 0.816, and HFNC-NBA 2.27 1/L, *p* = 0.244), deep breathing (median CPAP-NBA − 2.27 1/L, *p* = 0.378 and HFNC-NBA 4.55 1/L, *p* = 0.623), speaking (median CPAP-NBA 0 1/L, *p* = 0.0523 and HFNC-NBA 9.09 1/L, *p* = 0.0140), or coughing (median CPAP-NBA − 17.31 1/L, *p* = 0.587 and HFNC-NBA 1.92 1/L, *p* = 0.365). The results indicate that both CPAP and HFNC have no clinically meaningful impact on aerosol emission. Therefore, the use of CPAP or HFNC does not expose healthcare personnel to greater concentrations of aerosols when compared to normal breathing in healthy participants.

## Introduction

Utilisation of respiratory support therapies such as continuous positive airway pressure (CPAP) and high flow nasal cannula (HFNC) are integral in managing various respiratory conditions, such as obstructive sleep apnea, chronic obstructive pulmonary disease, respiratory infections, and acute respiratory distress syndrome^1–4^. Despite the benefits of these interventions, questions persist regarding their impact on emission of airborne particles and infection transmission^5–7^ because a variety of viruses, including influenza, SARS-CoV-2, and respiratory syncytial virus (RSV), can be transmitted through aerosols^8^.

Airborne particles are engaged in complex dynamics with the surrounding environment and can both shrink and grow depending on factors such as the relative humidity. In practice, 10 μm-size particles in still air take minutes to fall^9^. In turbulent indoor air with upward air streams, particles may remain suspended in air for extended periods, even if they are large enough to settle rapidly in still air^10^. In medical literature, particles < 5 μm have traditionally been considered aerosols due to their ability to reach distal parts of the airways in inhalation therapy^11^. On the other hand, ACE-2 receptors for the SARS-CoV-2 virus are found in the upper respiratory tract, making them accessible to larger particles as well^12^. Therefore, defining upper or lower size limit for potentially infectious particles is not straightforward.

Breathing aids alter the fluid mechanics of the airways by altering pressure or introducing an external source of airflow. CPAP delivers continuous positive pressure to maintain airway patency^13^ while HFNC provides high-flow oxygen-enriched air through nasal cannulas^2^, both potentially influencing respiratory dynamics and aerosol generation^14^. As a result, a significant airflow may occur over moist mucous membranes in the respiratory tract, where pathogens may reside^15–17^. Aerosols are generated during all human respiratory activities including normal breathing and speaking^15^. Medical procedures that increase particle production compared to normal breathing are classified as ‘aerosol generating’^18^. Notably, the World Health Organization (WHO) refrained from making a recommendation on the choice among HFNC, CPAP, and NIV in their guideline for COVID-19^19^, citing data uncertainty. In Finland, both CPAP and HFNC are deemed as aerosol generating procedures by the local authority Finnish Institute for Health and Welfare^20^. The amount and duration of aerosol exposure affects the likelihood of infection^21^. Therefore, personal protection should be used in procedures with high aerosol emission or long duration^18^.

In recent years, individual small studies have suggested that respiratory support treatments, such as non-invasive ventilation, do not generate more aerosol emissions than situations without respiratory support^22–24^. However, these studies have been small, and the level of evidence has not yet been sufficient to prompt changes in interpretations by organizations such as the WHO or local authorities. Our study aims to evaluate differences in both aerosol emissions and exposure with CPAP and HFNC compared to no breathing aid (NBA) with a systematic and controlled study design to support the assessment of the risk associated with respiratory support treatments in evaluating the airborne transmission risk of diseases.

## Materials and methods

### Participants

A total of 20 healthy participants were recruited for this study. The measurements were conducted between January and July 2023 at Helsinki University Hospital Ear-, Nose, and Throat Clinic. There were no inclusion criteria in this study. An acute or chronic respiratory disease was the only exclusion criterion. Four participants were excluded from the final analysis due to data corruption.

#### Setup

Measurements were performed in a laminar flow operating room (OR) with an air change rate of 30 times per hour, which provided practically particle free environment for the measurements. Participants laid on a surgical bed in a half-sitting Fowler position. Particle concentrations (particles/cm^3^) and particle size distributions (0.253–35.15 μm) were measured with a Grimm 11D Portable Aerosol Spectrometer (GRIMM Aerosol Technik GmbH & Co. KG, Ainring, Germany) using 31 size channels every 6 s at a flow rate of 1.2 L/min. Particles were categorised into three size groups: Small (< 1 μm), Medium (1–5 μm), and Large (> 5 μm). The categorisation was based on the aerodynamic diameter of particles, which dictates their behaviour in airstream^25^.

**Fig. 1:**
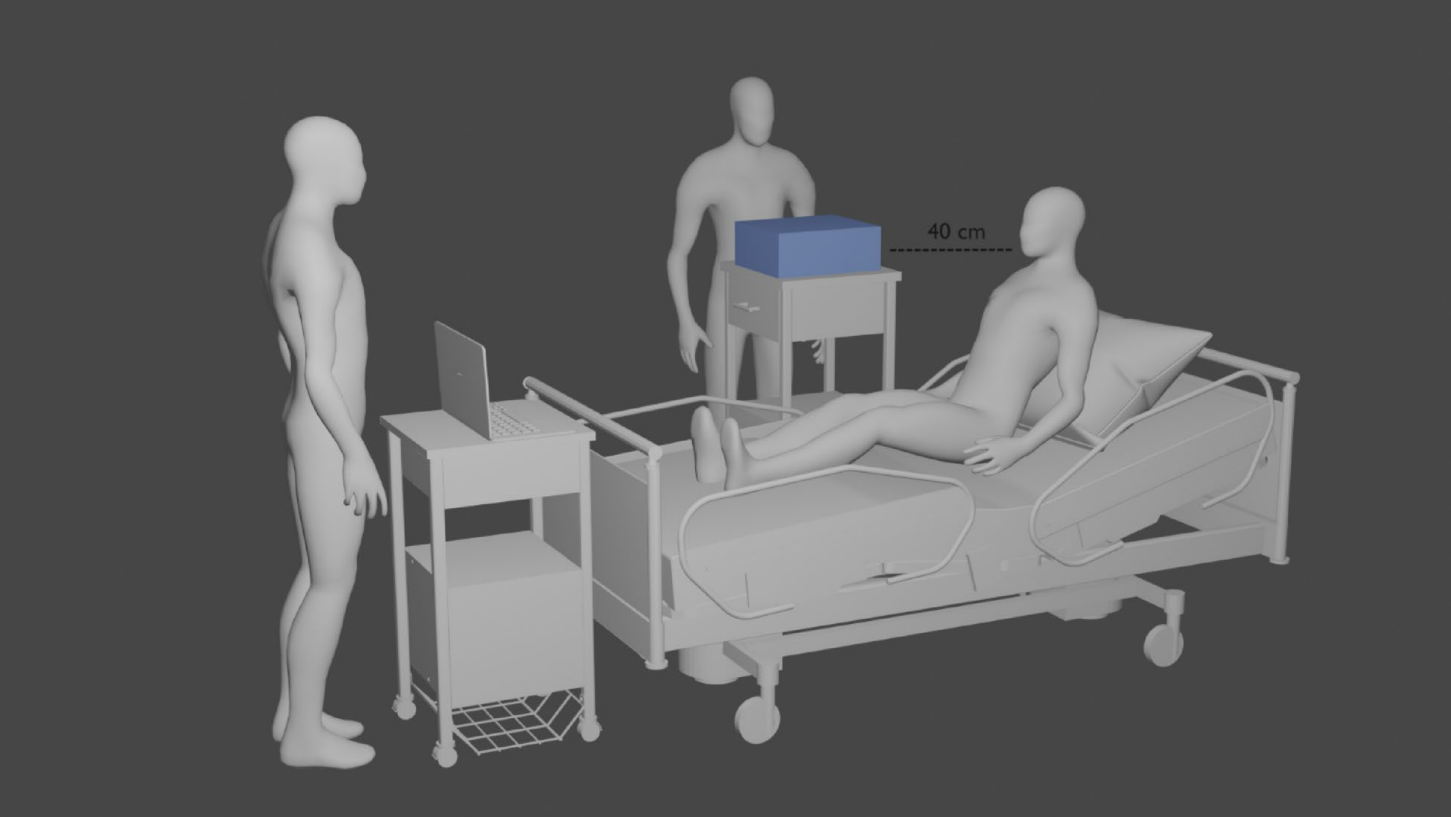
The experimental setup during measurements. The optical particle sizer (in blue) was placed 40 cm diagonally from each participant’s face.

The spectrometer was placed at a height of 120 cm and 40 cm diagonally from the participant’s face, as an estimated location of a healthcare worker’s face during care of a patient with CPAP or HFNC (see Fig. 1). The data collection started after the breathing aid was placed on the face of the participant.

#### Protocol

Background aerosol levels were measured in the OR separately for each participant. The measurement protocol consisted of 4 one-minute phases with one-minute breaks: normal breathing, deep breathing, speaking, and coughing (Table 1). Participants were instructed with a slideshow. The participants were not specifically instructed to keep their mouths open or closed in order to simulate a real clinical situation. The protocol was performed consecutively with no breathing aid (NBA), with CPAP (Oxylog^®^ 3000 Plus, Draeger, Lübeck, Germany), and with HFNC (Optiflow Thrive system, Fisher & Paykel Healthcare, Auckland, New Zealand). A full-face CPAP mask (NovaStar, Draeger, Lübeck, Germany) proportionate to each participant’s face was selected. CPAP was set to 5 mBar and 40% O_2_. HFNC was set to 50 L/min O2 at 34 °C.

**Table 1:** The instructions given to participants during each phase of a measurement protocol.

Action	Instructions
Normal breathing	Participants were instructed to look at a sample picture of a beach with no instructions on breathing, so as not to allow them to become conscious of breathing.
Deep breathing	Participants were instructed to breathe in tandem with a looping video of an expanding and contracting shape.
Speaking	Participants were instructed to read a shown text using their normal speaking voice.
Coughing	Participants were instructed to cough 3 times and then repeat that additionally twice with 30s breaks in between.

#### Statistical analysis

The Wilcoxon signed-rank test was used to assess statistical significance of observed differences in aerosol emissions between the non-invasive breathing aids (CPAP or HFNC) and the reference value (NBA). For all actions and breathing aids, the difference in particle emissions compared to normal breathing was calculated for each individual participant, and then the median of these differences was determined along with the range (minimum and maximum values). Statistical significance was assessed using the paired sample Wilcoxon signed-rank test. The Bonferroni-Holm method was used to control for type 1 error from multiple comparisons.

## Results

Of the sixteen healthy volunteers, nine (56%) were female and seven (44%) were male. The median age was 32.5 years (range 24–82). The detailed numerical results, including median, minimum, and maximum percentage changes for CPAP and HFNC compared to NBA, along with the corresponding p-values and adjusted p-values are presented in Table 2. We observed no statistically significant differences in aerosol emissions with CPAP and HFNC compared to NBA. While the initial p-value for HFNC was statistically significant (Table 2), this significance was lost after multiple comparison corrections.

**Table 2:** Median particle concentrations (range) during the different phases and corresponding p-values compared to NBA.

Action	Breathing aid	Particles/L (Median, Min-Max)	*p*-values
Breathing	NBA	18.18 (0.00-254.55)	
	CPAP	13.64 (0.00-77.27)	0.816
	HFNC	20.45 (4.55-309.09)	0.244
Deep Breathing	NBA	9.09 (0.00-172.73)	
	CPAP	6.82 (0.00-77.27)	0.378
	HFNC	13.64 (0.00-222.73)	0.623
Speaking	NBA	9.09 (0.00-127.27)	
	CPAP	9.09 (4.55-131.82)	0.0524
	HFNC	18.18 (9.09-313.64)	0.0140
Coughing	NBA	28.85 (3.85-107.69)	
	CPAP	11.54 (0.00-138.46)	0.587
	HFNC	30.77 (0.00-484.62)	0.365

The change in particle concentrations with CPAP and HFNC compared to NBA. NBA (no breathing aid), CPAP (continuous positive airway pressure), HFNC (high-flow nasal cannula).

**Fig. 2:**
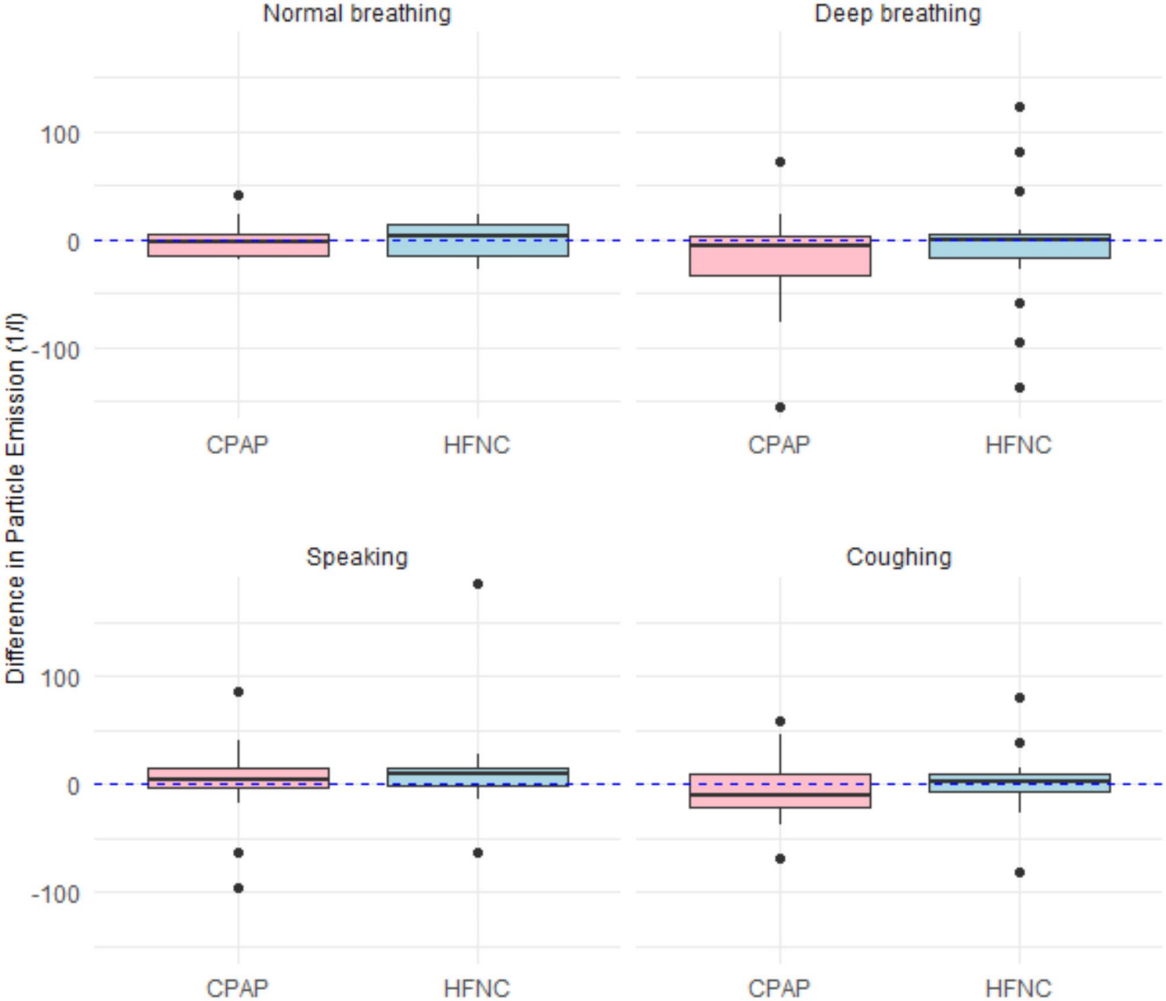
The differences in particle emissions between CPAP/NBA and HFNC/NBA during normal breathing, deep breathing, speaking, and coughing. The boxplots show medians, interquartile ranges, and total ranges excluding outliers. The dashed blue line at zero represents the baseline of no change compared to NBA. For graphical reasons, few outlying values were excluded from the visualisation: normal breathing HFNC (272.72731), normal breathing CPAP (-240.90914), normal breathing HFNC (-227.27277), coughing HFNC (403.84618).

Most of the particles detected were small (< 1 μm). Out of 192 measurements, small particles were found in 172 (89.58%). Medium size (1 -> 5 μm) particles were found in 82 (42,7%) of the measurements. The larger particles were only detected in eight measurements (0,042%) out of 192 (four during coughing, three during speaking and one during deep breathing). Larger size (> 5 μm) particles were not detected during normal breathing at all. Six participants (6/16, 37.5%) contributed 80% of the aerosol emission. Figure 2 shows the difference in particle emissions for CPAP and HFNC compared to NBA across the different activities. The zero line indicates no change in aerosol emissions relative to NBA.

## Discussion

In this study, we investigated aerosol generation in noninvasive respiratory support with CPAP or HFNC to assess the risk of airborne pathogen transmission. We did not observe any statistically significant differences in aerosol emissions between CPAP or HFNC compared to NBA during activities such as breathing, speaking or coughing. The finding reinforces the notion that, contrary to previous understanding, respiratory support treatments are not inherently significant aerosol-generating procedures.

Our results are in line with previous studies for both HFNC and CPAP, but also for positive pressure non-invasive ventilation (NIV)^22,24^. Similar results for HFNC have also been reported for patients with respiratory infection^23^. However, increased particle production when using breathing aid has also been reported, but the effect remained smaller than the difference between respiratory activities^24^.

In accordance with existing literature, the aerodynamic diameter of the particles observed in our study were mainly < 1 μm^26^. During coughing maneuvers, CPAP demonstrated a non-significant decrease in aerosol emissions compared to NBA, while HFNC showed a slight increase. Gaeckle et al. reported similar non-significant trend when using NIV, indicating that positive pressure and the tightly fitted face mask may help to limit the number of emitted particles^22^. Similarly, Pearce et al.. (2016)^27^ and Hamilton et al.. (2022)^18^ reported a reduction in aerosol emissions with CPAP compared to no intervention. Pearce et al. found a 15% reduction in smaller particles with CPAP use, and Hamilton et al. reported reduced aerosol generation even in the presence of large air leaks. A similar result was observed by Bem et al. (2021) in their study of high-flow nasal cannula (HFNC), where they found that the variation between subjects was more prominent than the effect of the intervention. In all the discussed studies, the individual variation was much larger than the differences between breathing aids. The clinical implication is that both HFNC and CPAP can be freely used, as the possible increase in infection risk is likely to be an order of magnitude smaller than the natural variation between patients.

The differences between these experimental setups were variations in measurement environments, ventilation conditions, and participant characteristics (healthy versus critically ill). Sample sizes across studies ranged from 10 to 25 participants, and experimental settings varied from controlled hospital settings to emergency departments.

There is ongoing debate and conflicting results about the concept of “super-spreaders” in the transmission of infectious diseases. It has been proposed that a small percentage (20%) of individuals are responsible for over 80% of the transmission of pathogens^28,29^. Likewise, in the present study, about 37.5% of participants contribute to 80% of the emissions. This suggests that the likelihood of aerosol-based transmission of respiratory infections is influenced by unique characteristics of the individual in addition to medical intervention. We also observed that some participants exhibited notably higher particle emissions than their counterparts during all studied activities. This aligns with the concept of “super-spreaders,” who disproportionately contribute to the spread of infectious diseases due to their higher particle emissions. Given this significant individual variation, it is important to treat all patients as potential super-spreaders, as it is not possible to identify these individuals in advance.

Our study was conducted in an operating room (OR) with a high air change rate, which presented both strengths and limitations. The high ventilation rate allowed us to perform the measurements in a virtually particle-free environment, minimising contamination from external sources. However, this environment also posed a significant limitation: it accelerates the removal of particles compared to a standard clinical setting. As a result, the particle concentrations reported in our study are unlikely to reflect the absolute values observed in less ventilated areas. Despite this limitation, the high ventilation setting enabled meaningful comparisons and interpretations of the relative effects of different breathing aids, which remains valuable.

We followed a standardised protocol, with specific instructions for each phase of the study (normal breathing, deep breathing, speaking, and coughing), ensuring consistency and reproducibility of results. The use of healthy volunteers further helped to standardise the baseline emission levels, making it easier to detect differences attributed to the devices tested. However, this choice also limits the generalisability of our results, as patients with respiratory conditions may exhibit different emission patterns^28^. Additionally, the sample size was relatively small, which reduced the statistical power to detect significant differences. Small sample sizes are a common challenge in aerosol research, given the complexity and resource-intensive nature of the measurements. Moreover, the aerosol distribution in our data exhibited a wide range, from zero emissions to very high values. This distribution, with a few “super spreaders” and many zero values, is difficult to model, presenting a challenge also encountered by other studies with similar datasets.

Finally, our measurements were taken from a distance representative of real-life exposure scenarios, without the use of a funnel. Some studies have employed funnels or close-proximity measurements to capture aerosol emissions more accurately^24^, but such methods may not reflect actual exposure conditions. By measuring at realistic distances, our study prioritised ecological validity, though this approach may have slightly reduced the precision of particle capture.

## Conclusion

In conclusion, the use of CPAP and HFNC does not seem to expose healthcare personnel to greater concentrations of aerosols when compared to normal respiratory activities in healthy participants. While no significant differences were observed in aerosol emissions during normal breathing, a non-significant decrease in emissions with CPAP during deep breathing and coughing maneuvers were observed. These findings contribute to our understanding of respiratory therapy-associated aerosol generation and have implications for infection control practices in healthcare settings.

## Data Availability

The clinical datasets generated and/or analysed during the current study are not publicly available due Finnish legislation on medical research, but are available from the corresponding author on reasonable request.
